# Bibliometric Analysis of Alpha-Synuclein Determination by Biopsy in Peripheral Tissues of Patients With Parkinson's Disease

**DOI:** 10.1155/ijta/6469893

**Published:** 2025-02-14

**Authors:** Aigerim B. Utegenova, Aigul P. Yermagambetova, Gulnar B. Kabdrakhmanova, Alima A. Khamidulla, Zhanylsyn U. Urasheva, Nazym K. Kenzhinа, Zhanna Zhussupova, Gulzhanat N. Nurlanova

**Affiliations:** ^1^Department of Neurology, West Kazakhstan Marat Ospanov State Medical University, Aktobe, Kazakhstan; ^2^The Course of Therapy, West Kazakhstan High Medicine College, Uralsk, Kazakhstan; ^3^Department of Neurology and Psychiatry, West Kazakhstan Marat Ospanov State Medical University, Aktobe, Kazakhstan; ^4^Department of Infectious Diseases, West Kazakhstan Marat Ospanov State Medical University, Aktobe, Kazakhstan

**Keywords:** alpha-synuclein, bibliometric analysis, biopsy, Parkinson's disease

## Abstract

**Study Evaluation:** The bibliometric analysis of the published results of the alpha-synuclein (*α*-syn) detection study in the peripheral tissues of patients with Parkinson's disease was carried out by us to study scientific activity and scientific productivity, to measure the quantitative and qualitative characteristics of scientific publications, author productivity, and citation of works, as well as the degree of interrelation between authors, journals, institutes, and countries.

**Purpose:** This study is aimed at bibliometrically analyzing the results of studies on the detection of *α*-syn in peripheral tissue biopsy of patients with Parkinson's disease from 2013 to 2023.

**Methods:** The data was extracted from the Scopus database collection using an inclusive search strategy. Performance analysis and science mapping were conducted using RStudio v.4.3.1 with the bibliometrix R-package. The analyzed data encompassed trends in publication and citation and the identification of leading institutions, primary sources, authors, and collaborative countries.

**Results:** A total of 124 relevant studies from 53 different sources were thoroughly analyzed. The analysis included the materials of 843 authors, which together gave an impressive average of 37.67 citations per document over the past decade. The annual growth rate in this area of research, according to calculations, was 10.84%, indicating a steady increase in the number of publications over the study period. The huge volume of research results is highlighted by the inclusion of 231 unique keywords of authors.

**Conclusion:** Studies on the detection of pathological *α*-syn in peripheral tissues for early morphological verification of the diagnosis of Parkinson's disease are relevant in clinical neurology. *α*-syn detected in the skin biopsy of patients on the basis of this study and data from the world literature meets the requirements for biomarkers.

## 1. Introduction

Alpha-synuclein (*α*-syn) is well-established as a genetic and pathogenic contributor to Parkinson's disease (PD), making it a promising candidate for biomarkers associated with the condition [1]. Operating initially as an unstructured protein, *α*-syn assumes diverse conformations influenced by its cellular environment. These varied forms are believed to exist in dynamic equilibrium in the brain under normal conditions, with this homeostasis undergoing changes in pathological states. While various forms of *α*-syn play roles in PD pathogenesis, the oligomeric form is notably the most toxic [2, 3].

The oligomerization of *α*-syn has been identified as preceding neuronal death in PD, underscoring its potential as a diagnostic tool for early disease detection. Various methods, including enzyme immunoassay (ELISA), Western blot, mass spectrometry, Luminex analysis, and seed amplification assays (SAAs), are employed to detect total *α*-syn as well as its oligomeric and phosphorylated pathological isoforms in body fluids such as cerebrospinal fluid, plasma, and saliva [4].

Recent insights indicating peripheral tissue deposition of *α*-syn prior to or simultaneously with brain accumulation have spurred investigations into visualizing disease-relevant *α*-syn aggregates in more accessible tissues for biopsy in routine clinical settings. Studies on *α*-syn levels in peripheral tissues, such as intestinal mucosa and salivary glands, for the differential diagnosis of PD from other synucleinopathies [5, 6] have yielded varied results, likely stemming from differences in sample preparation and analytical methods. This highlights the imperative for greater standardization to enhance the speed and reproducibility of data across independent laboratories [7].

A prospective approach involves a lifetime diagnosis through immunohistochemical (IHC) analysis of biopsies from peripheral tissues (e.g., sigmoid colon, skin, and submandibular salivary glands) to determine *α*-syn presence. Some researchers posit that identified *α*-syn in these peripheral tissues could serve as a biomarker for PD. However, challenges arise in histologically differentiating pathological and normal peripheral *α*-syn, attributed to factors such as variations in study methodologies, biopsy techniques, storage media, antibodies used for IHC studies, and the absence of independent blind replication across separate research centers. Addressing these complexities is vital for advancing reliable diagnostic methodologies in the study of PD. [8] In the updated criteria developed by the Movement Disorder Society Task Force from 2019, several innovative markers have been published with convincing and pathophysiologically plausible evidence that further supports the concept of prodromal PD. One of those of particular interest is tissue biopsy: Phosphorylated *α*-syn in skin biopsy has been shown to be sensitive (55%–100%), as well as highly specific (> 90%) in PD and prodromal PD. [9] The aim of the present bibliometric analysis was to study the results of studies on the detection of *α*-syn in peripheral tissue biopsy of patients with PD in the recent 10 years.

## 2. Methods

### 2.1. Search Results

**Figure 1:**
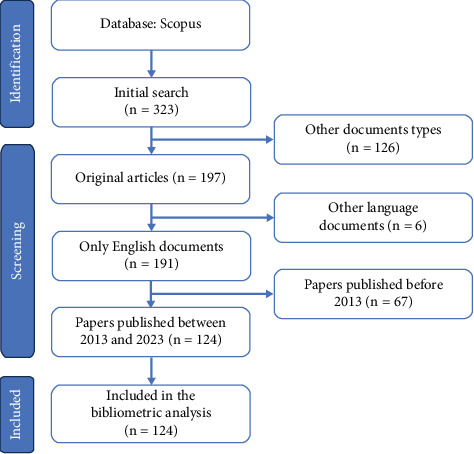
The flowchart of the screening process using PRISMA for bibliometric analysis of alpha-synuclein determination by biopsy in peripheral tissues of patients with Parkinson's disease between 2013 and 2023.

In November 2023, data were collected from the Scopus to comprehensively analyze research on *α*-syn determination by biopsy in peripheral tissues of patients with PD. The search strategy used in this study was aimed at being inclusive and covered various aspects of the topic. The search strategy employed was as follows: “biopsy” AND “Parkinson” (title) and “disease” AND “alpha-synuclein” (abstract). To ensure the accuracy and relevance of the data, specific inclusion criteria were applied. These criteria included the following: (1) articles published between 2013 and 2023; (2) articles written in English; and (3) exclusion of review articles, proceeding papers, book chapters, and editorial material. To provide a visual representation of the data extraction process, a Preferred Reporting Items for Systematic Reviews and Meta-Analyses (PRISMA) flowchart outlining the selection process is presented in Figure 1.

### 2.2. Performance Analysis

In this study, performance analysis and science mapping were conducted using specific software tools. RStudio v.4.3.1 with the bibliometrix R-package (http://www.bibliometrix.org; access date: 10 November 2023) was utilized for these analyses [10]. Biblioshiny, with its web features, was employed for data analysis. This software has the capability to function with a single database only. Scopus was chosen due to its provision of comprehensive and detailed citation information. This feature is particularly valuable for conducting thorough bibliometric analysis and assessing the impact of research outputs.

The local publication trends and average total citations per article were measured for each year. To identify the most productive journals, the number of publications was considered, and Bradford's law was applied to identify core journals, which are a few journals contributing significantly to citations in the field [11].

### 2.3. Identification of Leading Institutions, Sources, Authors, and Collaborating Countries

The top 10 most productive institutions and authors were ranked based on the percentage of papers they produced. Relationships between institutions and authors were visualized to understand their collaboration patterns. For country analysis, the percentage of articles from each country was used to rank the most productive country, and the percentage of multiple-country production was measured for the top 10 countries. The country collaboration network was mapped based on the number of publications produced by each country.

### 2.4. Keyword Frequency Analysis

Timeline analysis was performed to observe how frequently specific keywords appeared over the years. TreeMap was created to illustrate the distribution and prominence of the top 10 most frequently occurring keywords. Thematic analysis was conducted to identify the main trends and topics within the selected articles.

## 3. Results

### 3.1. Summary of Reports

The aim of this study was to comprehensively study the results of global studies on the detection of *α*-syn in peripheral tissue biopsies of patients with PD published in the period from 2013 to 2023. A total of 124 relevant studies from 53 different sources were thoroughly analyzed. The analysis included the materials of 843 authors, which together gave an impressive average of 37.67 citations per document over the past decade. The main conclusions concerning the detection of *α*-syn in peripheral tissue biopsy of patients with PD are presented on the basis of the most cited documents over the past decade in Table 1. In addition, the annual growth rate in this area of research, according to calculations, was 10.84%, indicating a steady increase in the number of publications over the study period. The huge volume of research results is highlighted by the inclusion of 231 unique keywords of the author.

### 3.2. Trends of Publication and Citation

**Figure 2:**
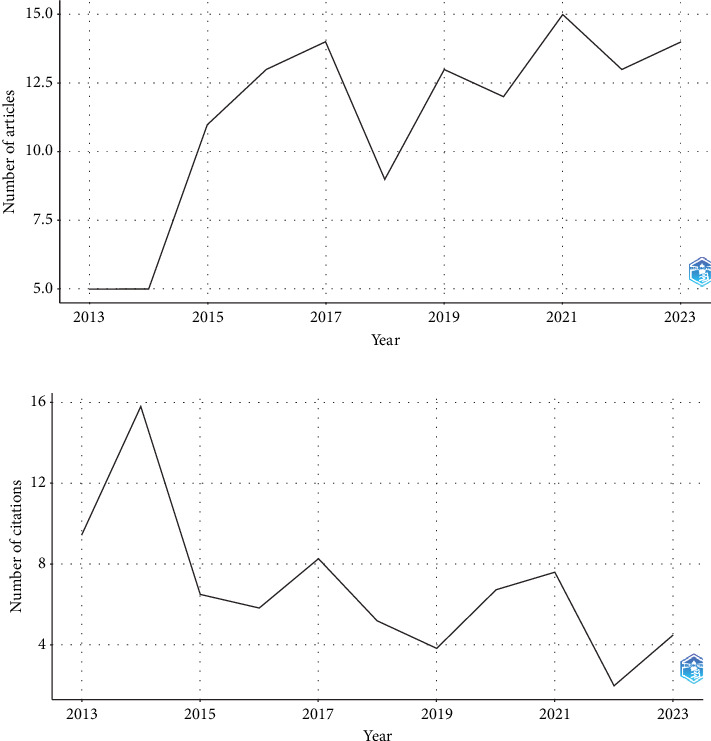
Global annual trend of (a) publication and (b) citation on the alpha-synuclein determination by biopsy in peripheral tissues of patients with Parkinson's disease (2013–2023).

The analysis revealed a significant increase in article production from 2013 to 2016, with a peak of over 15 articles in 2016. This period likely reflects a flourishing research environment, possibly due to increased funding or a surge in research interest and activity in specific fields. A dramatic decline was observed in 2017, where the number of articles fell below 5. The subsequent years displayed a recovery, with a return to previous levels in 2018 and a consistent pattern of fluctuation thereafter. The graph showed another peak in 2021, just below the 2018 level, followed by a slight decline and stabilization by 2023.  Figure 2 illustrates the trend of publications in recent years (Figure 2(a)) and the number of annual citations from 2013 to 2023 (Figure 2(b)).

**Figure 3:**
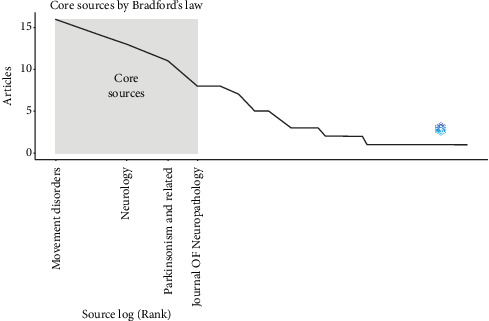
The plot of Broadford's law identified four core journals on the alpha-synuclein determination by biopsy in peripheral tissues of patients with Parkinson's disease (2013–2023).

Using Bradford's law, which describes the distribution of scientific articles between different journals, we identified four main journals that were recognized as the best choice for researchers (Figure 3). According to Bradford's law, these main journals together account for a significant part of the total number of articles published on the definition of *α*-syn in peripheral tissues in patients with PD. After analyzing the data on publications in these major journals, we noticed that the journal *Movement Disorders* became the most prolific, making a significant contribution to 16 articles, which is approximately 11.51% of the total number of articles for the study period. In addition, we investigated local citations obtained by these major journals from other articles in our dataset. It is noteworthy that *Movement Disorders* stood out with the largest number of local citations, collecting a total of 14 citations (Table 2).

### 3.3. Most Productive Authors, Institutions, Countries, and Their Collaboration Network

In the realm of PD research, particularly concerning *α*-syn determination by biopsy in peripheral tissues, the “University of Ulsan College of Medicine” emerges as the leading institution, contributing 15 articles to the literature. Following closely, the “Banner Sun Health Research Institute” holds the second-highest publication count with 14 articles. The “Universidad Autónoma de San Luis Potosi” has produced 12 articles, marking its significant contribution to this field.

Several esteemed institutions, including “Harvard Medical School,” “IRCCS Istituto Delle Scienze Neurologiche di Bologna,” “Li Ka Shing Knowledge Institute,” “Mayo Clinic,” “Mayo Clinic College of Medicine,” “Rush Medical College,” and “University of Toronto,” each have recorded nine articles. It is pertinent to note an apparent formatting issue in the data representation for the “University of Toronto,” where the label seems truncated and includes an HTML entity for an ampersand (&), suggesting a potential data extraction or labeling error.

**Figure 4:**
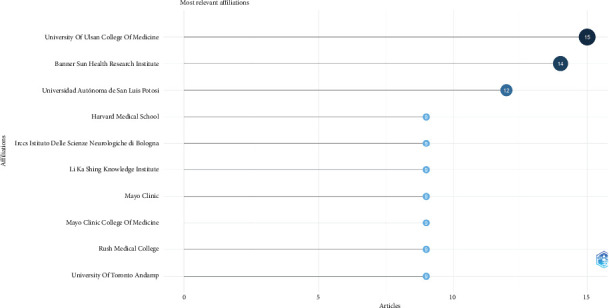
Most productive authors, institutions, countries, and their collaboration network.

In the graphical representation, as depicted in Figure 4, the size of nodes represents their publication volume as a visual cue for the number of articles, with larger size of nodes denoting higher publication counts. This visual approach aids in the immediate recognition of the most prolific institutions in this research area.

Among individual authors, Donadio V., Liguori R., and Beach T. are distinguished by their publication volume, contributing 16, 16, and 15 of the articles, respectively.

**Figure 5:**
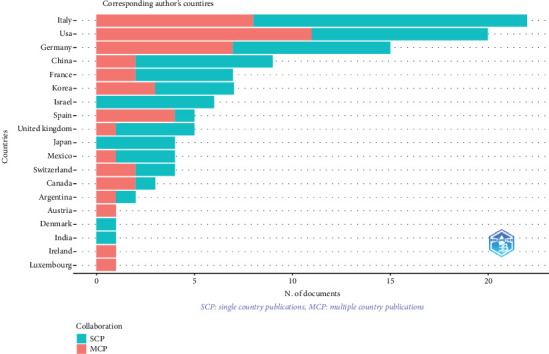
Leading publishing countries on the alpha-synuclein determination by biopsy in peripheral tissues of patients with Parkinson's disease (2013–2023). SCP: scientific collaboration pattern. MCP: most cited paper.

**Figure 6:**
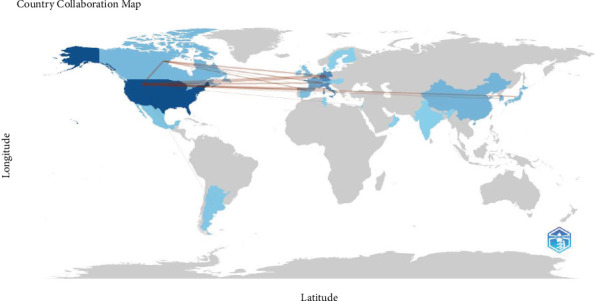
World collaboration map on the alpha-synuclein determination by biopsy in peripheral tissues of patients with Parkinson's disease (2013–2023). The intensity of color saturation corresponds to the increasing number of articles within each country.

Collaboration strength was mostly derived from the United States and European countries (Figure 5). A world collaboration map illustrating the *α*-syn determination by biopsy in peripheral tissues of PD patients (2013–2023) is presented. The map's color saturation intensity correlates with the number of published articles from each country, highlighting research contributions globally (Figure 6).

### 3.4. Co-Occurrence, Hotspots, and Emerging Keywords

The most commonly found author keywords were examined using Biblioshiny. The most common keywords of the authors were analyzed using Biblioshiny. The analysis covered common peripheral tissues, pathological proteins, and terms related to PD. The keywords “Parkinson's disease” and *α*-syn show an upward trend: 263 and 193 cases in 2023, respectively.

**Figure 7:**
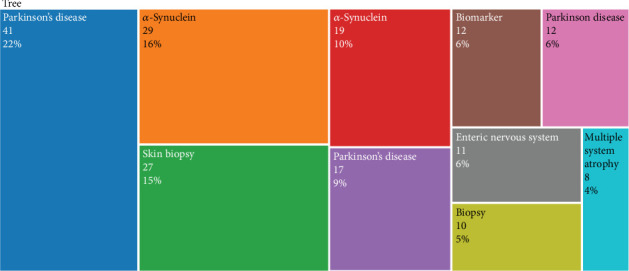
TreeMap representing top ten author's keywords in the research on the alpha-synuclein determination by biopsy in peripheral tissues of patients with Parkinson's disease (2013–2023).

The term “Adult” has achieved the highest cumulative frequency by 2023, exhibiting a marked and consistent upward trajectory. This indicates its increasing prevalence in scientific literature over time, reflecting a broader research focus on adult populations. Both “Humans” and “Human” demonstrate significant and steady increments in occurrences. This pattern likely mirrors the expansive scope of research encompassing human subjects or issues, underscoring the centrality of human-based studies in scientific inquiry. “Parkinson Disease” has experienced a notable escalation in cumulative mentions, particularly starting around 2013. The sharp increase observed circa 2016 and the continued growth thereafter may signify intensified research activities or a heightened volume of publications pertaining to PD. “Alpha-Synuclein,” closely associated with PD, follows a similar upward trend as “Parkinson Disease.” This parallel suggests a potential expansion in research or increased frequency of citation and discussion in the literature, specifically concerning this protein's role in the disease. Demographic terms such as “Male,” “Female,” “Aged,” and “Middle Aged” display a moderate yet gradual increase. This trend may reflect a sustained research interest in these demographic groups, highlighting their importance in various scientific studies. The term “Article” reveals a consistent upward trajectory, likely indicative of the growing volume of scientific articles being published or indexed. Such an increase can be attributed to the expansion of scientific inquiry and the proliferation of academic journals. Graphical representation of these trends offers valuable insights into the evolving landscape of scientific research. It enables the identification of emerging research areas, growing interests, and the impact of specific diseases and demographic factors on the scientific literature. The overall upward trend across all terms suggests a broadening in the volume of literature encompassing these varied topics. TreeMap depicts the top ten authors' keywords used in research on *α*-syn determination by biopsy in peripheral tissues of PD patients (2013–2023), providing insights into the primary research themes and trends in the field (Figure 7).

The temporal analysis of critical keywords in PD research uncovers notable trends and shifts in focus areas. In 2022, the emergence of the term “duodenum” suggests its newfound relevance or intensified research interest in this period. This may indicate a novel aspect of study or a paradigm shift in the field. The frequency of “synucleinopathy” has shown a progressive increase, culminating in a peak in 2022. This trend likely reflects an expanding research interest or output in this area, possibly due to advancements in understanding the role of synucleinopathies in neurodegenerative diseases. The term “genetics” has demonstrated a steady uptrend over the years. This consistent increase underscores the enduring and perhaps growing significance of genetic research in understanding and addressing PD and related conditions. “Parkinson's disease” and “alpha-synuclein” have maintained a prominent presence in recent research, suggesting their sustained relevance and potential escalation in scientific attention in recent years. This trend highlights the ongoing focus on the pathophysiology and therapeutic approaches targeting *α*-syn in PD. Demographic-related terms such as “aged,” “male,” “female,” and particularly “80 and over” (with a notable peak around 2019) imply a research inclination towards aging populations during this period. This could reflect the increasing importance of understanding PD in the context of an aging global demographic. Clinical and diagnostic terms like “cross-sectional study,” “diagnostic accuracy,” “polysomnography,” “major clinical study,” and “priority journal” signal their relevance in the research landscape. Their presence indicates a focus on methodological rigor and the significance of clinical findings in the field.

**Figure 8:**
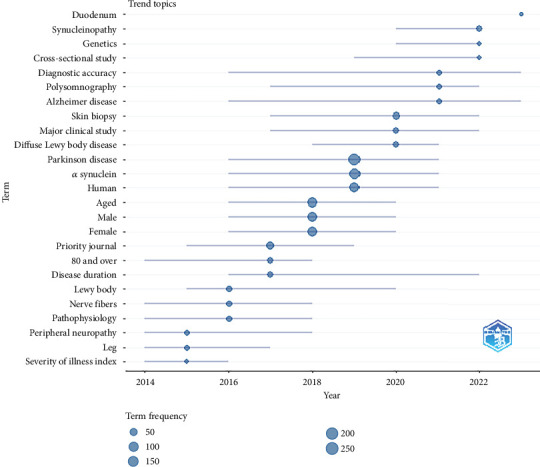
The timeline of the trend topics. Each bubble indicates the peak of frequency used for each, while the line indicates the years it was used.

Moreover, the inclusion of disease-specific terms such as “Alzheimer's disease,” “diffuse Lewy body disease,” and “peripheral neuropathy” suggests a broader scope of research interest, potentially indicative of increased scientific or clinical attention to these conditions (Figure 8).

In summary, this study comprehensively reviewed global research output on *α*-syn determination by biopsy in peripheral tissues of patients with PD over the past decade, identifying top journals; impactful articles; and collaborations between institutions, authors, and countries, as well as important and emerging keywords. The findings provide valuable insights into the research landscape and highlight potential areas for future studies.

## 4. Discussion

Bibliometric analysis offers a unique perspective on the trajectory and thematic evolution of research focused on the detection of *α*-syn in peripheral tissues of PD patients. This analysis facilitates the identification of key trends, influential authors, institutions, and nations contributing significantly to this domain. *α*-syn's role in neurodegenerative processes, particularly its involvement in neural inclusion formation, has garnered considerable interest in contemporary PD research. Through bibliometric assessment, one can discern the publication dynamics in this field, thereby gauging its scientific prominence and future research trajectories [12]. A notable outcome of such analysis is the evident escalation in interest regarding *α*-syn's presence in peripheral tissues of PD patients. This trend underscores the scientific community's growing recognition of peripheral tissues' role in elucidating PD mechanisms.

Furthermore, this analysis highlights the primary contributors to this research area, enabling the formation of collaborative networks and acknowledging individual scientists' contributions. Examination of keywords and thematic areas provides insights into the focal points of current research, guiding future studies towards promising subdomains.

However, bibliometric analysis is not without limitations. Relying solely on a single database might overlook pertinent publications. Additionally, publication quantity does not necessarily equate to research quality, and prevailing trends might occasionally reflect transient scientific fashions rather than substantive scientific progress. Globally, researchers are investigating for analyzing biopsies from various sites, such as the sigmoid colon, skin, and submandibular salivary glands, to detect *α*-syn for PD diagnosis. Histological differentiation between pathological and normal peripheral *α*-syn in biopsies presents challenges. These may stem from diverse research methodologies, biopsy site and depth variations, storage media, the use of nonspecific antibodies, and a lack of blind replication in independent studies [13–15]. Supported by the Michael J. Fox Foundation, the Fox Foundation for Parkinson's Research has conducted a large multicenter study since 2013, focusing on standardized detection methods for *α*-syn in peripheral tissues [16, 17].

### 4.1. Trend Analysis and Future Research Directions

The term “duodenum” saw a marked increase in usage in 2022, indicating a surge in research interest or publication activity in this area. This shift may signal new discoveries or therapeutic focuses in gastrointestinal research, meriting further exploration. The term “synucleinopathy” also experienced an upsurge, peaking in 2022. This aligns with the expanding literature connecting synucleinopathies with various neurodegenerative diseases, including PD. This trend may be attributable to advancements in diagnostic methods or an intensified focus on these conditions' molecular aspects. The consistent appearance of terms such as “genetics,” “Parkinson's disease,” and “alpha-synuclein” indicates sustained interest in these areas, emphasizing *α*-syn's central role in PD research.

Demographically related terms like “aged,” “male,” “female,” and “80 and over” suggest ongoing emphasis on demographic factors in research, likely due to their relevance in disease epidemiology, clinical trial design, and personalized medicine approaches. Clinical and diagnostic terms reflect a focus on research methodology and clinical finding reporting. The recurrent mention of “polysomnography” suggests continued interest in sleep studies, possibly linked to neurodegenerative diseases, sleep disorders, or respiratory conditions. The co-occurrence of “Alzheimer's disease” and “diffuse Lewy body disease” with “Parkinson's disease” indicates a broader interest in neurodegenerative disorders, potentially driven by the increasing aging population and the need to address these diseases' growing prevalence.

The temporal citation trend depicted in the graph offers insights into the scholarly impact of research *α*-syn in peripheral tissues in PD from 2013 to 2023. The peak in average citations per year in 2014 suggests heightened scholarly interest or significant articles published that year. From 2020, a rising trend indicates a resurgence in the domain's impact, influenced by previous research, emerging paradigms, or external factors. The increasing citations in recent years may reflect the typical citation lifecycle. Overall, the fluctuating yearly averages highlight the dynamic nature of research impact, emphasizing the need for longitudinal analyses. In summation, the delineated citation patterns across the preceding decade afford substantive insights into the field's progressive trajectory. These patterns reveal epochs of heightened research endeavors, directional shifts in scholarly focus, and the conceivable influence of extrinsic factors on the academic community's engagement and citations. A comprehensive bibliometric analysis would conventionally necessitate an expanded dataset encompassing publication volumes, context of citations, and the interrelations among the citing and cited works. The most prolific contributors to the field were Donadio and Liguori, with Beach having notable output as well, publishing 16 and 15 articles, respectively. The majority of their publications occurred during the period spanning 2020–2023. Donadio focused on the identification of phosphorylated *α*-syn in skin biopsies from individuals with neurodegenerative disorders, with particular emphasis on comprehensive linking of data. Donadio's seminal work, titled “Skin Nerve A-Synuclein Deposits: A Biomarker for Idiopathic Parkinson Disease,” published in the *Journal of Neurology* in 2014, holds a 54th percentile citation ranking [18]. Liguori's research, ranking second in productivity, centers around the Systemic Synuclein Sampling Study (S4), an innovative initiative aimed at establishing a reliable diagnostic and progression biomarker for PD. S4 comprehensively assesses *α*-syn in various tissues and biofluids within the same subject, providing crucial insights into optimal biomarker sources and the evolution of peripheral *α*-syn across the disease spectrum. According to Kuzkina et al., the aggregation of *α*-syn is considered a key histoneuropathological hallmark of PD. In recent years, *α*-syn depositions have been observed premortem in peripheral nerves across various tissues, including the easily accessible skin. Several studies have reported the IHC detection of phosphorylated *α*-syn in dermal nerve fibers [19]. Additionally, S4 correlates systemic *α*-syn profiles with objective measures of nigrostriatal dopaminergic function, enhancing our understanding of PD's pathophysiological progression [20, 21]. Beach et al., ranking third in output, contributed significantly to the field with research on the IHC detection of synuclein pathology in skin, specifically in cases of idiopathic rapid eye movement sleep behavior disorder and parkinsonism [16, 22].

Publication patterns reveal distinct strategies, with Italy displaying a notable inclination towards single-country productions, accounting for 14 publications. Conversely, the United States engaged in collaborative research, coauthoring 11 publications with researchers from various countries, including Canada, Germany, Switzerland, Spain, Italy, France, Korea, and China. Burst keywords, indicative of cutting-edge topics, encompass PD, *α*-syn, aged individuals, males, females, humans, middle-aged individuals, articles, adults, and humans. Remarkably, these keywords have not diminished in relevance over time; instead, their frequency of mention has surged significantly, underscoring their sustained significance in contemporary research discourse.

Overall, this analysis offers a roadmap for future research directions, highlighting emerging areas and foundational topics that continue to attract scientific interest. Our study is the first study to access on the detection of *α*-syn in peripheral tissues in PD in a bibliometric way. However, it is essential to acknowledge the limitations of this study, which are associated with the use of a single publication database. Scopus has covered many publications; however, some publications from databases, such as Web of Science, and PubMed, may not be included in this study. Due to our bibliometric analysis approach using empirical data from original articles, we focused on article metadata rather than content, extracting author details, institutions, and countries for productivity, collaboration, and impact assessment. Textual content analysis was not included. Additionally, the bibliometric approach relies on the accuracy and consistency of the metadata within the databases, introducing a degree of dependence on the quality of indexing and categorization. Furthermore, it should be noted that only English-language full-text articles were analyzed. Even if abstracts were available in English, such publications were excluded. As a result, certain articles from journals published in national languages might not have been included in the analysis.

## 5. Conclusions and Future Prospect

Over the past decade, research on the detection of *α*-syn in peripheral tissues in PD has shown a significant increase. Italy and the Unite States have made significant contributions to this field, as evidenced by their representation among the authors of 22 and 20 articles, respectively. Thus, the issue of lifetime diagnosis of PD remains relevant with the identification of a pathomorphological substrate with a high degree of reliability in peripheral tissues, especially the skin of patients whose biopsy is the least invasive and easily reproducible. *α*-syn detected in the skin biopsy of patients on the basis of this study and data from the world literature meets the requirements for biomarkers. In this regard, further multicenter studies are needed in order to unify and integrate approaches to the algorithm and protocol of IHC analysis of biopsy material of patients with PD.

### 5.1. Strengths of the Study

This study demonstrates robustness through its systematic application of bibliometric methodologies, comprehensively surveying diverse sources to afford a comprehensive perspective on the determination of *α*-syn in peripheral tissues through biopsy among PD patients. The analytical scope extends across a considerable timeframe, enabling the discernment of longitudinal trends and advancements. The inclusion of multiple parameters, such as authorship, citations, and keyword analysis, enhances the comprehension of the research landscape, furnishing valuable insights for prospective inquiries. The delineation of pivotal journals, prolific authors, and influential keywords establishes a solid groundwork for researchers and policymakers to navigate the dynamic domain of *α*-syn determination research in the context of PD pathogenesis.

## Figures and Tables

**Table 1:** The top 10 most cited documents on alpha-synuclein determination by biopsy in peripheral tissues of patients with Parkinson's disease (2013–2023).

**Rank**	**First author**	**Year**	**Journal**	**DOI**	**TCs**	**TCs per year**	**Normalized TC**
1	Wang N.	2013	*Neurology*	10.1212/WNL.0b013e3182a9f449	166	15.09	1.60
2	Cersosimo M.G.	2013	*J Neurol*	10.1007/s00415-012-6801-2	218	19.82	2.10
3	Doppler K.	2014	*Acta Neuropathol*	10.1007/s00401-014-1284-0	181	18.10	1.15
4	Hilton D.	2014	*Acta Neuropathol*	10.1007/s00401-013-1214-6	245	24.50	1.55
5	Donadio V.	2014	*Neurology*	10.1212/WNL.0000000000000316	219	21.90	1.39
6	Visanji N.P.	2015	*Neurology*	10.1212/WNL.0000000000001240	218	12.89	1.98
7	Sprenger F.s.	2015	*Neurology*	10.1212/WNL.0000000000002126	116	12.89	1.98
8	Donadio V.	2016	*Ann Neurol*	10.1002/ana.24567	114	14.25	2.45
9	Stolzenberg E.	2017	*J Innate Immun*	10.1159/000477990	116	27.00	3.27
10	Doppler K.	2017	*Acta Neuropathol*	10.1007/s00401-017-1684-z	181	25.86	3.13

Abbreviation: TCs, total citations.

**Table 2:** The top 10 most cited journals on the alpha-synuclein determination by biopsy in peripheral tissues of patients with Parkinson's disease.

**Sources**	**Articles**
*Movement Disorders*	14
*Neurology*	12
*Parkinsonism and Related Disorders*	11
*Journal of Neuropathology and Experimental Neurology*	7
*Journal of Parkinson's Disease*	7
*Annals of Clinical and Translational Neurology*	5
*Neuroscience Letters*	5
*NPJ Parkinson's Disease*	5
*Acta Neuropathologica*	3

## Data Availability

All data supporting the findings of this study are fully available without restriction. Researchers can access the data by contacting the corresponding author.
